# Preeclampsia as an early manifestation of cardiovascular–kidney–metabolic syndrome

**DOI:** 10.3389/fphys.2026.1796673

**Published:** 2026-04-14

**Authors:** Priscila Oliveira Barbosa, Luiz Sérgio Lima-Junior, Ricardo Carvalho Cavalli, Gizele Celante, Roberto da Silva Gomes, Valeria Cristina Sandrim

**Affiliations:** 1 Department of Gynecology and Obstetrics, Ribeirão Preto Medical School, University of São Paulo, São Paulo, Brazil; 2 Department of Pharmaceutical Sciences, College of Health and Human Sciences, North Dakota State University, Fargo, ND, United States; 3 Department of Biophysics and Pharmacology, Institute of Biosciences, São Paulo State University, Botucatu, São Paulo, Brazil

**Keywords:** cardiovascular disease, cardiovascular-kidney-metabolic syndrome, chronic kidney disease, metabolic syndrome, preeclampsia, pregnancy

## 1 Introduction

Recently, the American Heart Association published a scientific advisory introducing cardiovascular–kidney–metabolic (CKM) syndrome, defined as a framework to describe the complex and bidirectional interactions among cardiovascular disease (CVD), chronic kidney disease (CKD), and metabolic disorders that collectively contribute to adverse health outcomes across the lifespan (Ndumele et al., 2023). This novel conceptualization, which encompasses multiple organ systems, provides an opportunity to identify individuals at increased risk of developing CKM syndrome early in life, highlighting subclinical and early manifestations that may precede overt disease.

Sex-specific differences may play a central role in the development and progression of CKM syndrome (Ji et al., 2025). Women appear to have a greater vascular sensitivity to metabolic and inflammatory stressors, which may be reflected in distinct patterns of cardiometabolic risk (Ji et al., 2025). Pregnancy represents a critical physiological state in which this vascular vulnerability may be unmasked, as it imposes substantial hemodynamic, metabolic, and inflammatory demands on the maternal cardiovascular system (Williams, 2003). When vascular adaptation is maladaptive, pregnancy-related complications may emerge as early risk markers of future cardiometabolic and renal disease.

In this context, the incidence of hypertensive disorders of pregnancy (HDP) has increased over the past three decades and now affect more than 15% of pregnancies worldwide (Sun et al., 2025). Among HDP, preeclampsia has moved beyond obstetrics because of its association with future cardiometabolic events (Mosca et al., 2011). Preeclampsia is primarily a vascular disorder with multi-organ involvement, including the kidneys (Dimitriadis et al., 2023), and persistent hypertension remains a common postpartum manifestation. Although blood pressure usually returns to normotensive levels within two to four weeks postpartum (Ditisheim et al., 2018), long-term, women with prior preeclampsia face increased risks of heart failure, stroke, coronary heart disease, and CKD (Wu et al., 2017; Lailler et al., 2024).

The link between preeclampsia and features of CKM syndrome may be detectable even before the clinical manifestation of overt CVD or CKD. The pathophysiological connections between these conditions are compelling because both have been associated with overlapping vascular, inflammatory, and metabolic networks, including endothelial dysfunction, chronic inflammation, insulin resistance, oxidative stress, and systemic metabolic dysregulation (Kittelson et al., 2024). Furthermore, the increasing burden of obesity worldwide represents a shared risk factor for both CKM syndrome and preeclampsia and may partly account for their frequent co-occurrence, potentially reinforcing cardiometabolic risk across the life course. Therefore, we propose that preeclampsia may represent an early-life sentinel event that identifies women at increased risk for subsequent CKM syndrome. This idea challenges the traditional view of preeclampsia as a pregnancy-limited condition and underscores the relevance of early postpartum risk recognition and longitudinal prevention strategies.

## 2 Shared pathophysiological mechanisms

Preeclampsia is heterogeneous, and mechanistic emphasis likely differs across phenotypes. Early-onset and/or recurrent disease is more often characterized by severe placental dysfunction, angiogenic imbalance, and pronounced systemic endothelial activation, whereas late-onset disease frequently coexists with maternal cardiometabolic vulnerability including adiposity, insulin resistance, preexisting hypertension or masked vascular dysfunction (Roberts et al., 2021; Chaiworapongsa et al., 2024). These phenotypes may carry different postpartum trajectories: in some individuals pregnancy primarily reveals baseline susceptibility, while in others postpartum vascular, metabolic, or renal perturbations may be more persistent and clinically apparent. Throughout this section, we highlight where specific pathways may be more prominent across preeclampsia subtypes.

### 2.1 Endothelial dysfunction

Endothelial dysfunction is characterized by reduced nitric oxide (NO) bioavailability, increased vascular stiffness, and a shift toward vasoconstrictive, pro-inflammatory, and pro-thrombotic states (Roberts et al., 1989; Roberts, 1998). In preeclampsia, endothelial dysfunction originates at the placental level, driven by abnormal placentation and angiogenic imbalance, but frequently evolves into a systemic vascular disorder affecting multiple organs (Roberts, 1998; Viana-Mattioli et al., 2025).

Importantly, endothelial dysfunction observed in preeclampsia may not be fully resolved after delivery. Postpartum women with a history of preeclampsia demonstrate adverse arterial hemodynamic profiles consistent with vascular remodeling and a trajectory of cumulative vascular vulnerability (Melchiorre et al., 2011; Orabona et al., 2017; Kirollos et al., 2019). Moreover, evidence of an early vascular aging phenotype has been reported postpartum after preeclampsia, with more pronounced arterial stiffening and central hemodynamic abnormalities among women with severe, preterm, or recurrent disease (Werlang et al., 2023). Accordingly, women with a history of preeclampsia have a higher long-term risk of chronic hypertension (Voskamp et al., 2025) and major adverse cardiovascular events, including myocardial infarction, coronary heart disease, and stroke (Birukov et al., 2020; Garovic et al., 2020).

Postpartum endothelial and hemodynamic abnormalities also appear to vary by clinical phenotype. More pronounced arterial stiffness and central hemodynamic alterations have been reported after early-onset, whereas late-onset disease may more strongly reflect maternal cardiometabolic vulnerability that predates pregnancy (Stergiotou et al., 2013; Ziganshina et al., 2022). Overall, these data support endothelial dysfunction as a key mechanistic link through which preeclampsia identifies, and in some phenotypes may amplify long-term vascular vulnerability, contributing to later cardiometabolic and renal risk within the CKM framework. Future work should prioritize longitudinal designs that include pre-pregnancy vascular assessment and repeated postpartum measurements, enabling separation of baseline susceptibility from pregnancy-associated vascular injury and identifying which endothelial and hemodynamic markers best predict later clinical outcomes.

### 2.2 Angiogenic imbalance

Angiogenic imbalance is another central component of preeclampsia, although it remains unclear whether it is a cause or a consequence of impaired placentation. Inadequate remodeling of the spiral arteries leads to placental ischemia and hypoxia, stimulating the release of antiangiogenic factors (Staff et al., 2022). Circulating placental-derived factors, particularly an excess of antiangiogenic mediators such as sFlt-1 and reduced proangiogenic signaling PlGF, promote widespread endothelial activation and permeability changes, contributing to the characteristic clinical spectrum of hypertension, proteinuria, and end-organ involvement (Maynard et al., 2003; Nunes et al., 2025).

Interestingly, angiogenic imbalance is not unique to pregnancy: a broadly similar antiangiogenic pattern is described in endothelial dysfunction associated with diabetes, CKD, and heart failure, conditions also characterized by arterial stiffness and microvascular rarefaction (Di Marco et al., 2015; Fadini et al., 2019; Molina-Pérez et al., 2022). This convergence suggests that dysregulated angiogenic signaling may represent a shared pathway through which diverse clinical contexts amplify endothelial injury, even if the upstream triggers differ.

A key unresolved issue is whether angiogenic dysregulation has meaningful persistence beyond pregnancy or whether it functions primarily as a pregnancy-limited mediator with long-term risk driven by underlying cardiometabolic vulnerability. Although sFlt-1 levels decline rapidly after delivery, reports of postpartum elevations are inconsistent across cohorts and timepoints, and findings may differ by preeclampsia subtype (Wikström et al., 2008; Akhter et al., 2017). Angiogenic imbalance is most consistently observed in early-onset and placental-driven phenotypes, whereas late-onset disease may involve a relatively greater contribution from maternal cardiometabolic factors with a less extreme antiangiogenic profile (Chaiworapongsa et al., 2024). Outside pregnancy, higher sFlt-1 levels have been linked to vascular and renal disease, including atherosclerosis progression, CKD, and myocardial infarction (Di Marco et al., 2009, 2015; Shin et al., 2010). However, the field still lacks clarity on whether postpartum antiangiogenic activity is a causal contributor to later disease, a marker of persistent endothelial sensitivity, or a correlation of baseline risk that predates pregnancy.

### 2.3 Metabolic dysfunction

Preeclampsia frequently co-occurs with metabolic dysfunction, but the relationship is bidirectional and heterogeneous. Excess adiposity is common among individuals who develop preeclampsia, and pre-pregnancy body mass index (BMI) ≥ 30 kg/m² is consistently associated with higher risk, often alongside insulin resistance and dyslipidemia (Spaan et al., 2012; Li et al., 2021; Abdi et al., 2025). Yet, obesity is not a pre-requisite for preeclampsia, as the condition occurs across the BMI spectrum, and many affected individuals have no clear pre-pregnancy risk factors (Wheeler et al., 2022). Moreover, excessive gestational weight gain has been linked to a higher risk of preeclampsia (Fortner et al., 2009).

A phenotype-informed view helps reconcile these observations. Late-onset preeclampsia more often clusters with maternal cardiometabolic vulnerability such as higher adiposity, preexisting or masked hypertension, hepatic steatosis risk, and insulin resistance, supporting a “maternal cardiometabolic–driven” pathway in which pregnancy functions predominantly as a stress-test revealing an established risk trajectory (Chen et al., 2022). By contrast, early-onset disease is more tightly linked to placental dysfunction and severe endothelial disturbance (Rana et al., 2022); in these cases, postpartum metabolic abnormalities may still emerge, but their drivers may differ (e.g., inflammation, altered vascular function, renal involvement, and treatment-related weight retention), and the magnitude and pattern of metabolic persistence may not mirror late-onset disease (Chen et al., 2022). Recurrent preeclampsia likely represents a higher-risk subgroup across both pathways, consistent with stronger underlying predisposition and/or more severe disease biology (Brouwers et al., 2018).

Postpartum studies support clinically relevant metabolic signals after preeclampsia, including lower insulin sensitivity and a higher prevalence of metabolic syndrome, with some evidence suggesting a stronger signal after early-onset disease (Hooijschuur et al., 2019; Akdemir et al., 2020). Central adiposity, in particular, has been associated with worse vascular and cardiometabolic profiles postpartum (Barbosa et al., 2025), aligning with the CKM syndrome in which dysfunctional adiposity and insulin resistance are early-stage features. However, it remains unresolved whether postpartum metabolic risk reflects (i) persistence of prepregnancy vulnerability unmasked by pregnancy, (ii) pregnancy-associated shifts in weight trajectory and insulin sensitivity, or (iii) phenotype-specific combinations of both.

Mechanistically, insulin has direct vascular actions, including stimulation of endothelial NO production and modulation of endothelin-1 (Scioscia et al., 2014). In insulin-resistant states, impaired endothelial insulin signaling reduces NO bioavailability and promotes a more vasoconstrictive, pro-inflammatory endothelial phenotype, changes that can further aggravate the endothelial dysfunction central to preeclampsia and plausibly contribute to long-term cardiovascular risk (Scioscia et al., 2014). Dyslipidemia adds another reinforcing layer, as a pro-atherogenic lipid pattern, including increased triglycerides and a predominance of small, dense LDL, has been reported in subsets of patients with preeclampsia (Spracklen et al., 2014; Hosier et al., 2023). From a CKM perspective, these metabolic changes are best interpreted as early risk signals whose persistence and trajectory likely differ across preeclampsia phenotypes. Longitudinal studies with pre-pregnancy metabolic phenotyping, pregnancy subtype classification (early vs late onset; placental- vs maternal-driven; recurrent vs isolated), and repeated postpartum metabolic assessments will be essential to determine which metabolic markers most strongly stratify later CKM outcomes and to identify the optimal windows for intervention.

### 2.4 Renin-angiotensin-aldosterone dysregulation

Dysregulation of the renin-angiotensin-aldosterone system (RAAS) is another key alteration in preeclampsia (Tsikouras et al., 2025). During normal pregnancy, there is a physiological attenuation of the vascular response to angiotensin II; in preeclampsia, however, heightened vascular reactivity to angiotensin II is observed, with increased sensitivity and signaling through the angiotensin II type 1 (AT1) receptor (Tsikouras et al., 2025). Beyond blood pressure regulation, AT1 activation promotes oxidative stress, endothelial activation, and vascular inflammation through mechanisms such as NADPH oxidase, reduced NO bioavailability, and upregulation of pro-inflammatory mediators (Nickenig and Harrison, 2002). RAAS signaling has also been implicated in the angiogenic imbalance characteristic of preeclampsia, including increased expression and release of antiangiogenic factors such as sFlt-1 and reduced proangiogenic signaling, thereby linking RAAS activation to placental dysfunction and systemic endothelial injury (Verdonk et al., 2014).

Clinically, RAAS overactivation contributes to the characteristic hypertension and sodium/fluid retention of the syndrome, while also reinforcing microvascular dysfunction across organs (Vogt et al., 2025). In the kidney, enhanced AT1 signaling and aldosterone excess may exacerbate glomerular endothelial injury and proteinuria, creating a biological bridge to later CKD (Xia et al., 2007). Importantly, although the removal of the placenta resolves the acute syndrome, increased sensitivity to angiotensin II can persist postpartum in women with prior preeclampsia, potentially contributing to long-term hypertension and cardiovascular risk (Saxena et al., 2010).

### 2.5 Genetic and epigenetic mechanisms

Emerging evidence has pointed out the shared genetic susceptibility and epigenetic programming between preeclampsia, CVD and CKD, suggesting these conditions appear to arise from overlapping genetic architecture and heritable predispositions that become clinically manifest during the physiological stress of pregnancy (Sitras et al., 2015). Family history represents one of the most consistent risk factors for preeclampsia, with familial clustering extending beyond reproductive outcomes to include essential hypertension, type 2 diabetes, and cardiovascular disease (Skjaerven et al., 2005). Genome-wide association studies have identified overlapping genetic loci between preeclampsia and cardiometabolic traits, including variants near *FLT1* (encoding sFlt-1), genes regulating the renin-angiotensin system (*AGT*, *ACE*, *AGTR1*), and endothelial nitric oxide pathways (*NOS3*) (McGinnis et al., 2017; Steinthorsdottir et al., 2020; Pereira et al., 2024). Recent polygenic risk score analyses demonstrate that genetic liability for CVD is elevated in women who develop preeclampsia, reinforcing a shared inherited vulnerability (Honigberg et al., 2023).

Beyond DNA sequence variation, epigenetic modifications provide an additional molecular link between preeclampsia and long-term cardiometabolic risk. Altered DNA methylation patterns, which influence genes implicated in angiogenesis, inflammation, and vascular remodeling, have been documented in placental tissue and maternal blood vessels during preeclampsia (Yeung et al., 2016; Cirkovic et al., 2020); furthermore, certain alterations appear to persist postpartum, with a recent study demonstrating differential methylation in offspring years after preeclampsia exposure, indicating a potential transmission and persistence of epigenetic modifications (Ross et al., 2025). Circulating microRNA profiles are also dysregulated in preeclampsia, with several miRNAs targeting pathways involved in endothelial function and vascular homeostasis (Lopes et al., 2025). Consequently, these epigenetic modifications affect processes central to both preeclampsia and CKM syndrome.

## 3 Preeclampsia as a CKM phenotype

The cardiometabolic perturbations initiated during preeclampsia persist long after delivery (Behrens et al., 2017; Hildebrand et al., 2017). For many women, preeclampsia represents the first clinical recognition of underlying cardiometabolic fragility. While some women enter pregnancy with overt risk factors, others appear metabolically healthy by conventional measures (Eastabrook et al., 2018); however, these may carry latent genetic or placental susceptibility. Pregnancy, as described above as a natural metabolic stress test (Williams, 2003), reveals subclinical insulin resistance, impaired lipid metabolism, and endothelial vulnerability that would have remained silent for years outside of gestation. In this light, preeclampsia functions as a sentinel clinical event within the CKM framework, often representing the earliest point at which cardiometabolic–renal vulnerability becomes clinically apparent. Rather than implying a single causal pathway, this framing emphasizes that pregnancy can expose heterogeneity in baseline susceptibility and risk trajectories. Consistent with this view, postpartum cardiometabolic and vascular risk markers are frequently detectable within months to years after an affected pregnancy.

**Figure 1:**
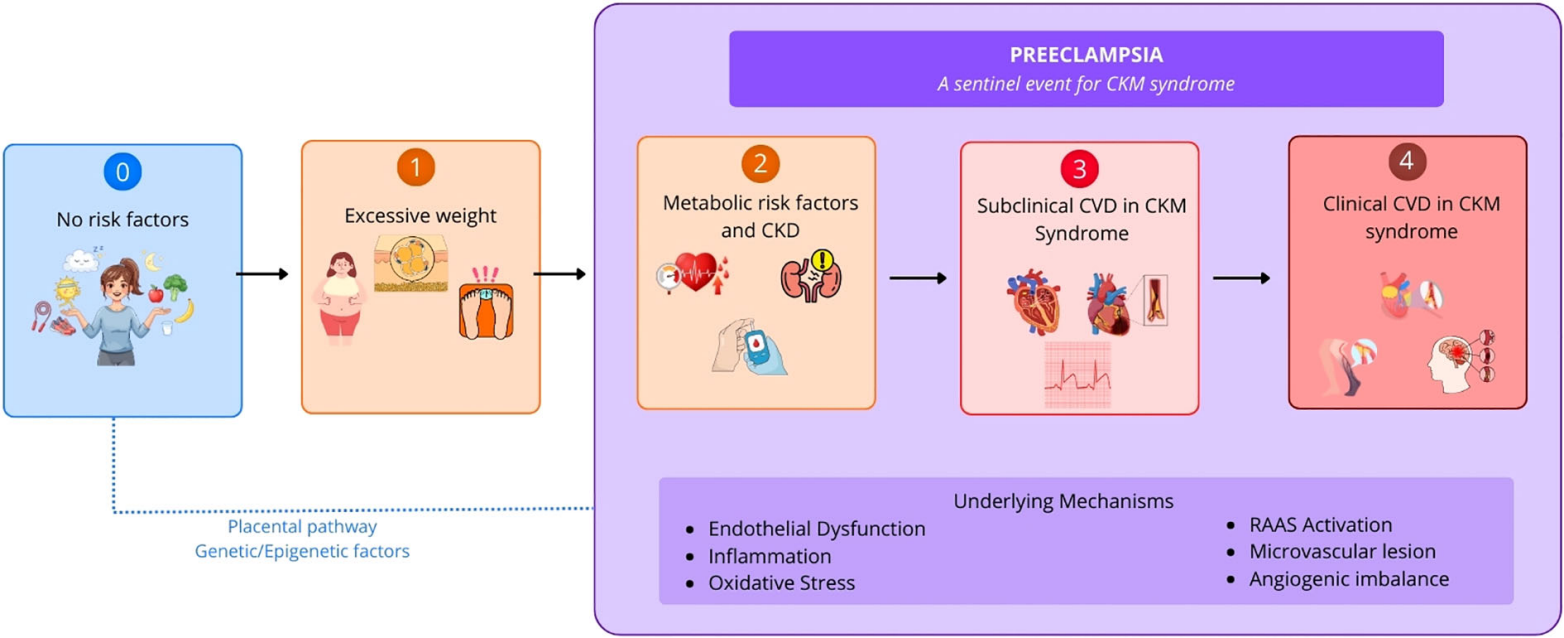
Conceptual idea of preeclampsia as a sentinel event in Cardiovascular-Kidney-Metabolic (CKM) syndrome. Preeclampsia overlaps CKM stages 2–4, functioning as a vascular stress test that unmasks pre-existing subclinical cardiometabolic vulnerability. Women without overt metabolic risk factors (stage 0) may harbor latent genetic or placental susceptibility that becomes clinically apparent during pregnancy, often manifesting as early-onset disease. Women with excess adiposity and dysfunctional adiposity (stage 1) frequently carry baseline endothelial dysfunction, insulin resistance, and chronic low-grade inflammation that remain clinically silent until the hemodynamic demands of pregnancy expose the underlying pathology. The mechanisms sustaining this vulnerability, including oxidative stress, angiogenic imbalance, persistent endothelial dysfunction, and metabolic dysregulation, are not resolved by delivery, explaining why postpartum risk markers such as hypertension, albuminuria, and dyslipidemia emerge within months to years after an affected pregnancy. Preeclampsia thus conceptually identifies women with features spanning stages 2 (metabolic risk factors), 3 (subclinical CVD), and 4 (symptomatic CVD) of the CKM syndrome. CKM, Cardiovascular-Kidney-Metabolic; CVD, Cardiovascular Disease; CKD, Chronic Kidney Disease.

Women with a preeclampsia history demonstrate sustained insulin resistance, elevated fasting glucose, dyslipidemia, and increased visceral adiposity years postpartum (Barry et al., 2015; Akdemir et al., 2020; Barbosa et al., 2025). The AHA CKM framework describes a continuum from Stage 0 (no CKM risk factors) through Stage 1 (excess/dysfunctional adiposity), Stage 2 (metabolic risk factors and/or CKD that elevate CVD risk), Stage 3 (subclinical CVD), and Stage 4 (clinical CVD) (Ndumele et al., 2023). As illustrated conceptually in **Figure 1**, preeclampsia can be positioned as a sentinel event that often brings CKM vulnerability to clinical attention and may cluster with features spanning Stages 2–4. Postpartum metabolic, vascular, and kidney risk markers, such as persistent hypertension, insulin resistance, dyslipidemia, albuminuria, and adverse hemodynamic profiles, may be detectable within months to a few years after an affected pregnancy and can be described within this staging continuum (Facca et al., 2018). Preeclampsia thus may represent an early clinical manifestation of pathways that also drive CKM progression. Therefore, rather than viewing metabolic risk factors as merely predisposing to preeclampsia, we should consider whether preeclampsia is itself the first metabolic event, the initial clinical presentation of a CKM trajectory in women.

## 4 Clinical implications

Despite strong evidence that HDP, particularly preeclampsia, is associated with an approximately two- to three-fold increase in long-term cardiovascular risk and earlier onset of cardiometabolic dysfunction, postpartum care remains a missed prevention opportunity in many clinical settings. Women with a history of preeclampsia develop hypertension, diabetes, dyslipidemia, and other components of cardiometabolic risk almost a decade earlier than those with normotensive pregnancies, yet structured follow-up and intervention strategies are inconsistently implemented across care pathways (Janssen et al., 2025). This underscores the imperative of structured cardiometabolic follow-up starting soon after pregnancy and continuing lifelong, rather than limiting assessment to the obstetric period.

Postpartum follow-up recommendations after preeclampsia vary across professional societies in timing, care ownership (obstetrics vs primary care vs cardiology), and the breadth of cardiometabolic testing as summarized recently (Garovic et al., 2022). Nonetheless, there is broad agreement on several core elements including early postpartum blood pressure assessment, transition to longitudinal care, lifestyle optimization, and periodic cardiovascular risk screening. An early review focused on blood pressure (and symptoms) is warranted within first 1–2 weeks after delivery for women with a history of preeclampsia, followed by a structured assessment at the routine postpartum visit (approximately 6–8 weeks) to evaluate blood pressure, body weight/adiposity measures, glucose regulation, and kidney markers (e.g., creatinine/eGFR and persistent proteinuria/albuminuria) as initial indicators of sustained risk. Additional assessment of fasting lipids and a more detailed metabolic profile can be performed at approximately 6 months postpartum and then periodically (often annually), both to detect emerging dyslipidemia and insulin resistance and to inform long-term prevention planning.

Lastly, traditional cardiovascular risk algorithms have not fully integrated HDP or CKM syndrome phenotypes, even though preeclampsia reflects early manifestations of shared vascular, metabolic, and renal pathophysiology that elevate lifetime disease risk (Inversetti et al., 2024). Priority populations for CKM-oriented follow-up include women with early-onset preeclampsia, those with severe features or concomitant adverse pregnancy outcomes, individuals with preexisting cardiometabolic conditions such as gestational diabetes or obesity, and those with persistent postpartum hypertension or proteinuria beyond 6–8 weeks. Emerging frameworks such as the American Heart Association’s CKM staging model further emphasize the interconnected progression of cardiometabolic and renal dysfunction across the life course, reinforcing the need to incorporate reproductive history into risk stratification paradigms. Incorporating pregnancy history into cardiovascular risk models could improve early identification of high-risk women and enable more personalized and timely prevention strategies. Moreover, care models must shift from obstetric discharge to longitudinal care planning, with structured interdisciplinary integration of cardiology, nephrology, endocrinology, and obstetrics to manage overlapping risks and prevent progression to overt CKM syndrome. The complexity and bidirectional nature of these intersecting disorders underscore the need for CKM-oriented prospective clinical trials in post-preeclampsia populations to define optimal screening intervals, risk thresholds, and therapeutic strategies tailored to this unique and frequently under-recognized high-risk group.

## 5 Conclusion

Preeclampsia should be reconceptualized not merely as an obstetric complication, but as a sentinel cardiometabolic event – an early clinical manifestation of vulnerability that predicts increased risk of CKM-related outcomes later in life. Placental stress and angiogenic imbalance, coupled with endothelial dysfunction, RAAS-mediated vascular injury, and metabolic derangements, may leave a persistent vascular–renal imprint that helps explain the excess burden of hypertension, CKD, and CVD later in life. Treating preeclampsia history as a CKM risk marker should motivate earlier postpartum screening, kidney-focused follow-up, and targeted cardiometabolic prevention, especially in women with severe, early-onset, or recurrent disease.
